# PB.29: Ultrasound-guided vacuum-assisted percutaneous excision of breast papillomas: results of long-term follow-up

**DOI:** 10.1186/bcr3529

**Published:** 2013-11-08

**Authors:** AJ Maxwell, G Mataka, S Whiteside

**Affiliations:** 1 Royal Bolton Hospital, Bolton, UK; 2 Nightingale Centre and Genesis Prevention Centre, University Hospital of South Manchester, UK; 3 Breast Unit, Royal Lancaster Infirmary, Lancaster, UK; 4 Department of Medical Statistics, University Hospital of South Manchester, UK

## Introduction

The purpose was to determine the frequency of recurrent/residual papillomas in women who had previously undergone vacuum-assisted excision biopsy (VAB) of benign papillomas without atypia and to identify factors that may be associated with recurrence.

## Methods

Women who had undergone VAB of papillomas and had subsequent breast imaging were identified from hospital records. Papilloma size, VAB device used and number of cores was recorded and subsequent imaging reviewed. Possible associations between the likelihood of recurrent/residual papilloma and patient age, lesion size and excised volume to papilloma volume ratio were analysed using Mann-Whitney U tests.

## Results

Thirty-four women had subsequent imaging available. Median follow-up was 1,052 days. Twelve women (35%) had evidence of a mass at the excision site, with a median time to identification of recurrent/residual papilloma of 1,369 days. In three of the six cases that underwent ultrasound the recurrence was larger than the original lesion. Three recurrences were removed at open surgical biopsy and one by further VAB. None of the 34 women were diagnosed with atypia or malignancy during follow up. No significant association was found between the risk of recurrence and the age of the patient, the size of the initial lesion or the ratio of the volume of tissue removed to the lesion volume.

## Conclusion

Recurrent/residual papillomas are common but most do not require further intervention. No definite risk factors were identified. VAB remains the excision method of choice for most benign papillomas without atypia.

